# Impact of Post-Operative Infection after CABG on Long-Term Survival

**DOI:** 10.3390/jcm12093125

**Published:** 2023-04-25

**Authors:** Agnieszka Zukowska, Mariusz Kaczmarczyk, Mariusz Listewnik, Maciej Zukowski

**Affiliations:** 1 Department of Infection Control, Regional Hospital Stargard, 73-110 Stargard, Poland; a.zukowska@op.pl; 2 Sanprobi Sp. Z o.o. Sp.k., 70-204 Szczecin, Poland; 3 Department of Cardiac Surgery, Pomeranian Medical University, 70-111 Szczecin, Poland; 4 Department of Anesthesiology, Intensive Care and Acute Intoxication, Pomeranian Medical University, 70-111 Szczecin, Poland

**Keywords:** infections, pneumonia, sepsis, CABG, long term survival, cardiac surgery

## Abstract

Coronary artery bypass grafting (CABG) is one of the most common cardiac surgical procedures. It is commonly known that post-operative infection has a negative impact on the patient’s short-term treatment outcomes and long-term prognosis. The aim of the present study was to assess the impact of perioperative infection on 5-year and 10-year survival in patients undergoing elective on-pump CABG surgery. The present prospective observational study was carried out between 1 July 2010 and 31 August 2012 among patients undergoing cardiac surgery at our centre. Infections were identified according to the ECDC definitions. We initially assessed the incidence of infection and its relationship with the parameters analysed. We then analysed the effect of particular parameters, including infection, on 5-year and 10-year survival after surgery. We also analysed the impact of particular types of infection on the risk of death within the period analysed. The significant risk factors for reduced survival were age (HR 1.05, CI 1.02–1.07), peripheral artery disease (HR 1.99, CI 1.28–3.10), reduced LVEF after surgery (HR 0.96, CI 0.94–0.99), post-operative myocardial infarction (HR 1.45, CI 1.05–2.02) and infection (HR 3.10, CI 2.20–4.28). We found a strong relationship between post-operative infections and 5-year and 10-year mortality in patients undergoing CABG. Pneumonia and BSI were the only types of infection that were found to have a significant impact on increased long-term mortality after CABG surgery.

## 1. Introduction

Coronary artery bypass grafting (CABG) is one of the most common cardiac surgical procedures. Despite developments in interventional cardiology offering non-surgical options to treat myocardial ischaemia, approximately 400,000 CABG procedures are performed each year worldwide [1,2]. Both the short- and long-term outcomes of CABG are good. One study based on data from the SWEDEHEART registry showed that of the 37,520 patients who underwent first-time CABG surgery in Sweden between 2006 and 2017, 2.8% (1057) did not survive the first 6 months after surgery [3]. A 2022 meta-analysis based on data from 180,459 patients undergoing CABG showed that the median overall survival of patients undergoing CABG with multiple arterial grafting was 17.54 years, whereas the median overall survival of patients undergoing CABG with single arterial grafting was 11.63 years [4]. It should be noted that a number of perioperative factors may affect short- and long-term prognosis and survival in patients undergoing CABG. Independent risk factors for long-term mortality include age, diabetes, atrial fibrillation, impaired left ventricular function, peripheral vascular disease, prior cerebrovascular accident and left main stem coronary disease [5,6,7,8]. It is commonly known that post-operative infection has a negative impact on the patient’s short-term treatment outcomes and long-term prognosis [9,10]. The majority of studies to date have assessed the impact of perioperative infection on short-term mortality (usually reported as 30-day mortality), showing that patients with post-operative infection have significantly increased mortality [11,12,13]. There are few studies in the literature that have investigated the impact of post-operative infection on long-term survival in patients undergoing cardiac surgery. Moreover, the study populations in the available research on the subject are generally heterogonous, including patients undergoing a number of different surgical as well as interventional cardiology procedures [14,15,16]. Furthermore, the studies have different follow-up periods, ranging from 2 to 10 years. The aim of the present study was to assess the impact of perioperative infection on 5-year and 10-year survival in patients undergoing elective on-pump CABG surgery.

## 2. Materials and Methods

The present prospective observational study was carried out between 1 July 2010 and 31 August 2012 among patients undergoing cardiac surgery at our centre. All the operated patients were prospectively entered into a clinical register, in which demographic data were anonymised and supplemented with data from the perioperative period that were collected until day 30 post-surgery. Of the 2640 patients operated on in the period analysed, we identified 780 patients who underwent elective, isolated on-pump CABG surgery. All patients with proven or suspected infection in the pre-operative period were not included in the study. The pre-operative protocol for patients before elective CABG included the removal or treatment of all potential sources of infection prior to admission. The pre-operative data included the following: age, sex, weight, height, comorbidities, left ventricular ejection fraction (LVEF), NYHA functional class, EuroSCORE and logistic EuroSCORE. The intraoperative data included the following: the duration of extracorporeal circulation, duration of the surgery, use of pressor amines and need for intra-aortic balloon pump (IABP) support. The post-operative data included the following: LVEF on the second day after surgery, the volume of post-operative drainage, volume of blood products transfused, duration of mediastinal drainage, CK-MB levels at 6, 12 and 24 h after surgery, duration of in-hospital stay, percentage of ICU (Intensive Care Unit) admission, and percentage of various complications, such as myocardial infarction, stroke, atrial fibrillation, infection and in-hospital mortality. Post-operative infections were defined as those that occurred within 14 days of surgery, excluding infections acquired during ICU stay. Infections were identified according to the ECDC definitions. These included the following: bloodstream infections (BSI), pneumonia, as well as sternal and limb surgical site infections (SSI). All antimicrobial therapy was based on ESCMID and IDSA guidelines, and empiric therapy was optimized after the culture results in all cases.

Information on 5-year and 10-year survival following surgery was obtained using the National Register of Cardiac Surgeries. We initially assessed the incidence of infection and its relationship with the parameters analysed. We then analysed the effect of particular parameters, including infection, on 5-year and 10-year survival after surgery. We also analysed the impact of particular types of infection on the risk of death within the period analysed.

For statistical analysis, survival curves were created using the Kaplan–Meier method. The Cox regression model was used to quantify the effect size. It was used to fit both univariable and multivariable regression models. The effect size was expressed as a hazard ratio (HR) with 95% confidence intervals. All variables that were significant in the univariable analysis were included in the multivariable analysis. Survival analysis was conducted using the survival package in R (https://cran.r-project.org accessed on 28 March 2023). Statistical significance was set at *p* < 0.05.

## 3. Results

A total of 780 patients were included in the study, with 189 women (24.2%) and 591 men (75.8%). Their mean age was 64.8 ± 8.8 years. In total, 42.1% (329) of the patients were active smokers at the time of surgery and 31.9% (252) had diabetes. All descriptives for the survivors and non-survivors data are shown in Table 1.

In total, 8.7% (69) of patients experiences post-operative infections. Pneumonia was the most common type of infection (36 patients (4.6%)), followed by BSI (15 patients (1.9%)), sternal wound infection (15 patients (1.9%)) and limb SSI (3 patients (0.4%)). A comparison of the parameters of patients with and without infection is shown in Table 2.

We found that patients who experienced post-operative infection were older, had a worse NYHA class and were significantly more likely to have type 2 diabetes and post-operative myocardial infarction. Moreover, they had a higher EuroSCORE, a higher logistic EuroSCORE and a lower pre-operative and post-operative LVEF. In total, 86.9% (685) of the patients included in the study were alive 5 years after surgery and 71.1% (560) were alive 10 years after surgery. A comparison of the parameters of patients who survived and those who did not survive at 10 years post-operation is shown in Table 3.

**Figure 1:**
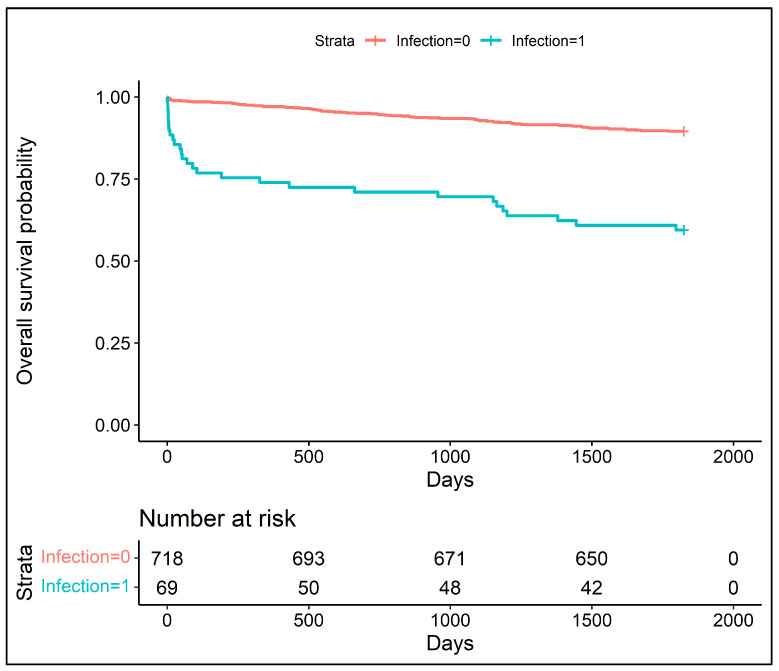
The Kaplan–Meier survival plot for patients with and without infections in 5 years observational period.

**Figure 2:**
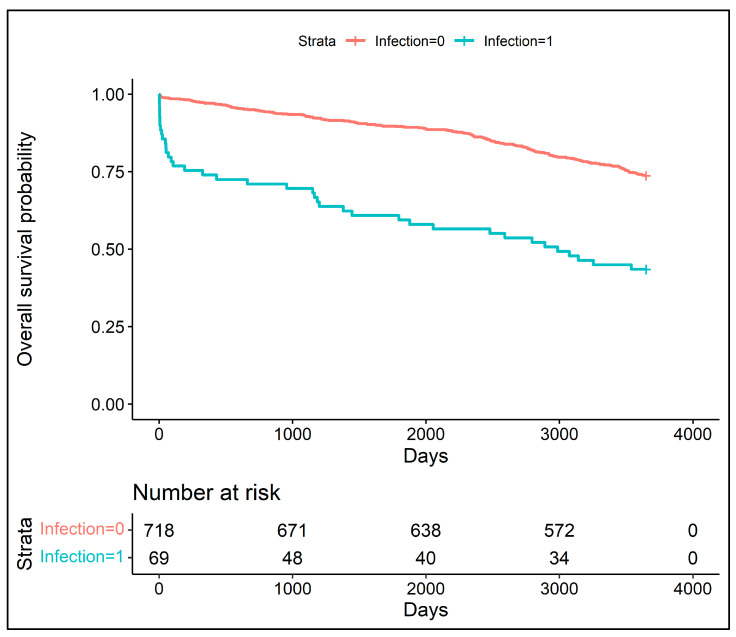
The Kaplan–Meier survival plot for patients with and without infections in 10 years observational period.

Patients who survived were younger and were less likely to suffer from or have suffered from myocardial infarction, stroke, diabetes, renal failure and peripheral artery disease. They had a lower EuroSCORE, a lower logistic EuroSCORE and higher pre-operative and post-operative LVEF. Moreover, they were significantly less likely to have experienced infection. The Kaplan–Meier survival plot demonstrated an increased 5-year mortality (28%) for patients with post-operative infection (Figure 1) and an increased 10-year mortality (52%) for the same group (Figure 2).

**Figure 3:**
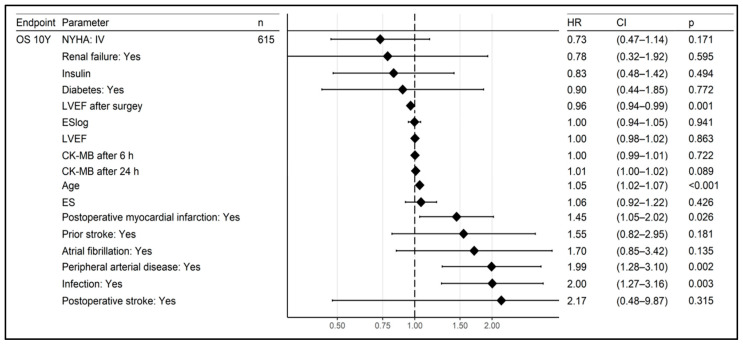
Risk factors for 10-year mortality in logistic regression.

The risk factors for 10-year mortality in the Cox logistic regression analysis are shown in Figure 3.

**Figure 4:**
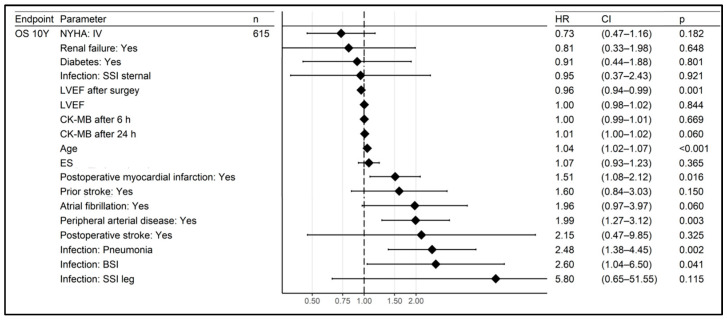
Risk factors for 10-year mortality, including the type of infection, in logistic regression.

The significant risk factors for a reduced survival were age (HR 1.05, CI 1.02–1.07), peripheral artery disease (HR 1.99, CI 1.28–3.10), reduced LVEF after surgery (HR 0.96, CI 0.94–0.99), post-operative myocardial infarction (HR 1.45, CI 1.05–2.02) and infection (HR 2.00, CI 1.27–3.16). A similar relationship between reduced survival and infection was also found for the 5-year follow-up period. When analysing the clinical presentations of infections, it was found that pneumonia (HR 3.91, CI 2.54–6.04) and BSI (HR 3.48, CI 1.78–6.79) had the strongest impact on increased mortality out of all the infection types studied. No similar significance was found for superficial SSI and DSWI. The risk factors for the 10-year mortality in the Cox logistic regression analysis, including the type of infection, are shown in Figure 4.

## 4. Discussion

The aim of the present study was to assess the impact of post-operative infection on long-term prognosis in patients undergoing on-pump CABG surgery. The demographic characteristics of the patients included in the study, such as age, sex, weight and smoking status, did not differ from the demographic data reported by other authors [3,17,18]. A comparison of patients with and without infection showed that patients with infection were older, were more likely to have diabetes, stroke, atrial fibrillation and pre-operative myocardial infarction, and had lower LVEF and a worse NYHA class. Diabetes is an independent risk factor for post-operative infection, which has also been confirmed by our findings [19,20]. All the other listed factors are indicative of a worse overall health and more advanced cardiovascular disease. A history of myocardial infarction or stroke indicates advanced atherosclerosis, whereas reduced LVEF, a higher NYHA class and atrial fibrillation indicate worse cardiac function. Therefore, we believe that the increased incidence of ICU admissions was, in most cases, the result of acquiring infections in the post-operative period, rather than infections resulting from ICU stays, as these were excluded from the study. In their study based on the data of 7352 patients from the European multicentre coronary artery bypass grafting (E-CABG) registry, Biancari et al. found that BMI, diabetes and atrial fibrillation are risk factors for DSWI in patients undergoing CABG surgery [21]. In contrast, in a study by Mancone et al., which analysed the incidence of post-operative infection after bypass surgery in 897 patients included in the SYNTAX trial, a high BMI was found to be the only significant predictor of major post-operative infection [20]. The incidence rates of particular types of infection in our study were similar to those reported by other authors. The overall incidence of infection in the patients included in the present study was 8.7%, with approximately half of infections being pneumonia, which is consistent with data from other centres. A study by Hadaya et al., based on data from the Nationwide Readmissions Database, showed that of the 444,165 patients undergoing elective CABG or valve operations, 8% experienced post-operative hospital acquired infections [22]. With regard to post-operative pneumonia, a 2018 study by Brescia et al., based on data from the Society of Thoracic Surgeons Adult Cardiac Surgery Database, showed that of the 324,085 patients undergoing CABG, almost 3% had post-operative pneumonia [23]. The incidence of DSWI in the patients included in the present study was 1.9%. In a study by Tatsuishi et al., which included 53,186 patients who had undergone thoracic cardiovascular surgery, the incidence of DSWI was similarly low, namely 1.43%. However, in that study, the in-hospital mortality of patients with DSWI was 24.7% [17]. The incidence of BSI in the patients included in the present study was 1.9%. In a study by Hadaya et al., the incidence of this type of infection was 2.4% [22].

In the present study, 86.9% (685) of patients were alive 5 years after surgery, whereas the 10-year mortality was 28.9%. The 5-year mortality in patients undergoing cardiac surgery ranges between 8.2% and 11.4% [15,24,25]. According to the literature, the significant factors that affect the long-term prognosis in patients undergoing CABG include age, sex, COPD, peripheral vascular disease, diabetes, renal failure, reoperation, post-operative LVEF, myocardial infarction, post-operative bleeding and infection [15,26]. Our multivariable analysis showed that the following were independent risk factors for an increased 10-year mortality in the patients undergoing CABG included in the present study: older age, peripheral artery disease, reduced post-operative LVEF, post-operative myocardial infarction and post-operative infection. The other listed parameters were significant only in the univariable analysis. In the case of renal failure, this may be due to the small number of patients with this condition. Patients undergoing reoperation were excluded from the present study. Our findings that pertain to the effect of the factors analysed in terms of the long-term prognosis of patients undergoing CABG surgery are consistent with the results previously reported by other authors. To date, the impact of perioperative infection on 5-year and 10-year survival has not been clearly identified. It seems logical that post-operative infections in patients undergoing serious surgery worsen prognosis. However, there are a limited number of reports on the subject in the literature. In 2015, Robich et al. published their analysis of the effect of post-operative infection on long-term survival based on 30,414 patients undergoing cardiac surgery. The study showed that despite successful post-operative treatment, cardiac surgery patients who experienced infections remained at a higher risk of death after approximately 1 year compared to patients who did not experience post-operative infections [27]. In their subgroup analysis of the SYNTAX Extended Survival trial, which was an extended follow-up of the international SYNTAX trial (multi-centre, open-label, randomised, controlled trial carried out in 85 centres in Europe and the USA), Ono et al. found that despite the association between perioperative infections and 5-year mortality in the patients included in the SYNTAX trial who underwent CABG—a finding which is consistent with our findings—there is no association between post-operative infections and 10-year mortality in these patients. This contrasts with our findings, which demonstrated a significant relationship between perioperative infections and 10-year mortality after CABG surgery [Ono]. In the SYNTAX trial, the number of patients in the CABG group was 897, which is similar to the number of patients included in the present study. The key difference between our study and the analysis by Ono et al. was the way in which data about infection were collected. In the study by Ono et al., the data were collected retrospectively, whereas the patients included in the present study were screened prospectively, which may explain the difference in the findings. It should be noted that the findings from the present study and those from the analyses relating to the SYNTAX trial were similar with respect to the impact of perioperative infections on 5-year mortality. However, a relationship between perioperative infections and 10-year mortality in patients undergoing CABG was observed only in the present study. Moreover, in the present study, pneumonia and BSI were the only types of infection that were found to have a significant impact on increased long-term mortality after CABG surgery. Authors should discuss the results and how they can be interpreted from the perspective of previous studies and of the working hypotheses. The findings and their implications should be discussed in the broadest context possible. Future research directions may also be highlighted.

This study also has several limitations. First, this is a single-hospital study. The use of administrative hospital databases introduced an inherent bias that should be taken into consideration. The time of ICU ventilation was not recorded, and we were thus unable to add it to statistical analysis. We also do not control for post-hospitalization confounders that might have a causal relationship with long-term mortality, as this is limited by our data sources.

## 5. Conclusions

We found a strong relationship between post-operative infections and 5-year and 10-year mortality in patients undergoing CABG. Pneumonia and BSI were the only types of infection that were found to have a significant impact on increased long-term mortality after CABG surgery.

## Figures and Tables

**Table 1:** A comparison of the parameters of survivors and non-survivors.

Parameter	Survivors *n* = 560	Non-Survivors *n* = 228	*p*
Female (*n*. %)	138/560 (25%)	51/228 (22%)	0.8974
Age (years. mean ± SD)	63 (9)	69 (8)	<0.0001
Weight (kg. mean ± SD)	81 (13)	80 (14)	0.6957
LVEF (%. mean ± SD)	51 (10)	46 (12)	0.0001
Insulin-dependent diabetes (*n*. %)	6/553 (1.1%)	2/228 (0.9%)	1
Insulin-non-dependent diabetes (*n*. %)	159/553 (29%)	92/228 (40%)	0.0134
Prior stroke (*n*. %)	18/553 (3.3%)	19/228 (8.3%)	0.0178
Myocardial infarction (*n*. %)	169/552 (31%)	96/228 (42%)	0.0143
Atrial fibrillation (*n*. %)	12/553 (2.2%)	14/228 (6.1%)	0.0352
NYHA functional class (*n*. %)			0.0170
1	4/542 (0.7%)	0/226 (0%)	
2	122/542 (23%)	28/226 (12%)	
3	290/542 (54%)	146/226 (65%)	
4	126/542 (23%)	52/226 (23%)	
ES (mean ± SD)	3.36 (2.48)	5.13 (2.81)	<0.0001
ES log (mean ± SD)	3.9 (4.2)	7.2 (6.8)	<0.0001
Peripheral arterial disease (*n*. %)	43/553 (7.8%)	44/228 (19%)	<0.0001
Renal failure (*n*. %)	13/553 (2.4%)	21/228 (9.2%)	0.0004
CPB time (minutes. mean ± SD)	50 (15)	50 (15)	0.8031
Operation time (minutes. mean ± SD)	153 (56)	150 (26)	0.8031
LVEF day after surgery %	53 (9)	47 (11)	<0.0001
CK-MB after 6 h	37 (20)	43 (38)	0.5946
CK-MB after 12 h	42 (32)	53 (70)	0.2007
CK-MB after 24 h	44 (35)	59 (100)	0.1307
Intraaortic balloon pump (*n*. %)	2/548 (0.4%)	2/227 (0.9%)	0.98331
Milrinone (*n*. %)	11/541 (2.0%)	9/222 (4.1%)	0.48196
TIA (*n*. %)	5/551 (0.9%)	0/227 (0%)	0.63631
Post-operative myocardial infarction (*n*. %)	6/551 (1.1%)	7/227 (3.1%)	0.27267
Post-operative stroke (*n*. %)	2/551 (0.4%)	5/227 (2.2%)	0.13234
Post-operative atrial fibrillation (*n*. %)	119/551 (22%)	58/227 (26%)	0.55622
Infection (*n*. %)	30/559 (5.4%)	39/228 (17%)	<0.0001
Infection source (*n*. %)			<0.0001
0	529/559 (95%)	189/228 (83%)	
BSI	6/559 (1.1%)	9/228 (3.9%)	
SSI sternal	9/559 (1.6%)	6/228 (2.6%)	
SSI leg	2/559 (0.4%)	1/228 (0.4%)	
Pneumonia	13/559 (2.3%)	23/228 (10%)	
Post-operative drainage (mL. mean ± SD)	552 (353)	530 (306)	0.9020
Red blood cells (mL. mean ± SD)	124 (271)	181 (326)	0.0363

LVEF—left ventricular ejection fraction, ES—EuroSCORE, ES log—logistic EuroSCORE, NYHA—New York Heart Association, TIA—transient ischemic attack, CPB—cardiopulmonary by-pass, CK-MB—creatine kinase-myocardial band, BSI—blood stream infection, SSI—surgical site infection.

**Table 2:** A comparison of the parameters of patients with and without infection.

Parameter	Infection (−)	Infection (+)	*p*
Female (*n*. %)	176/718 (25%)	13/69 (19%)	0.6571
Age (years. mean ± SD)	65 (9)	68 (9)	0.0051
Weight (kg. mean ± SD)	81 (13)	84 (13)	0.06332
LVEF (%. mean ± SD)	50 (11)	46 (12)	0.0313
ES (mean ± SD)	3.71 (2.60)	5.60 (3.12)	<0.0001
ES log (mean ± SD)	4.5 (4.7)	8.3 (9.2)	<0.0001
Insulin-dependent diabetes (*n*. %)	7/713 (1.0%)	1/67 (1.5%)	1
Insulin-non-dependent diabetes (*n*. %)	218/713 (31%)	32/67 (48%)	0.0411
Prior stroke (*n*. %)	33/713 (4.6%)	4/67 (6.0%)	1
Myocardial infarction (*n*. %)	242/712 (34%)	23/67 (34%)	1
Atrial fibrillation (*n*. %)	22/713 (3.1%)	4/67 (6.0%)	0.6571
Hypertension (*n*. %)			
NYHA functional class (*n*. %)			0.02625
1	4/700 (0.6%)	0/67 (0%)	
2	145/700 (21%)	5/67 (7.5%)	
3	400/700 (57%)	36/67 (54%)	
4	151/700 (22%)	26/67 (39%)	
Peripheral arterial disease (*n*. %)	75/713 (11%)	12/67 (18%)	0.3156
Renal failure (*n*. %)	29/713 (4.1%)	5/67 (7.5%)	0.6458
Intraaortic balloon pump (*n*. %)	2/709 (0.3%)	2/65 (3.1%)	0.1683
Milrinone (*n*. %)	11/696 (1.6%)	9/66 (14%)	<0.0001
TIA (*n*. %)	4/711 (0.6%)	1/66 (1.5%)	1
Post-operative myocardial infarction (*n*. %)	8/711 (1.1%)	5/66 (7.6%)	0.0074
Post-operative stroke (*n*. %)	6/711 (0.8%)	1/66 (1.5%)	1
Post-operative atrial fibrillation (*n*. %)	160/711 (23%)	16/66 (24%)	1
CPB time (minutes. mean ± SD)	50 (14)	52 (18)	0.8494
Operation time (minutes. mean ± SD)	151 (50)	157 (33)	0.1689
LVEF day after surgery %	52 (10)	45 (11)	<0.0001
Chest drainage time (hours. mean ± SD)	29 (15)	31 (17)	0.9266
CK-MB after 6 h	37 (21)	56 (59)	0.0373
CK-MB after 12 h	42 (33)	71 (113)	0.0212
CK-MB after 24 h	45 (38)	82 (163)	0.0068
Post-operative drainage (mL. mean ± SD)	544 (334)	568 (403)	0.8015
Red blood cells (mL. mean ± SD)	130 (278)	251 (366)	0.0058
Time of in-hospital stay (days mean ± SD)	7.2 (1.7)	13.7 (13.4)	0.0001
ICU admission (*n*. %)	4/718 (0.6)	20/69 (28.9)	<0.0001
In-hospital mortality (*n*. %)	4/718 9(0.6)	8/69 (11.6)	<0.0001

LVEF—left ventricular ejection fraction, ES—EuroSCORE, ES log—logistic EuroSCORE, NYHA—New York Heart Association, TIA—transient ischemic attack, CPB—cardiopulmonary by-pass, CK-MB—creatine kinase-myocardial band, ICU—Intensive Care Unit.

**Table 3:** A comparison of the parameters of patients who survived and those who did not survive at 10 years post-operation.

Parameter	Survivors *n* = 560	Non-survivors *n* = 228	*p*
Female (*n*. %)	138/560 (25%)	51/228 (22%)	0.8974
Age (years. Mean ± SD)	63 (9)	69 (8)	<0.0001
Weight (kg. mean ± SD)	81 (13)	80 (14)	0.6957
LVEF (%. Mean ± SD)	51 (10)	46 (12)	0.0001
Insulin-dependent diabetes (*n*. %)	6/553 (1.1%)	2/228 (0.9%)	1
Insulin-non-dependent diabetes (*n*. %)	159/553 (29%)	92/228 (40%)	0.0134
Prior stroke (*n*. %)	18/553 (3.3%)	19/228 (8.3%)	0.0178
Myocardial infarction (*n*. %)	169/552 (31%)	96/228 (42%)	0.0143
Atrial fibrillation (*n*. %)	12/553 (2.2%)	14/228 (6.1%)	0.0352
NYHA functional class (*n*. %)			0.0170
1	4/542 (0.7%)	0/226 (0%)	
2	122/542 (23%)	28/226 (12%)	
3	290/542 (54%)	146/226 (65%)	
4	126/542 (23%)	52/226 (23%)	
ES (mean ± SD)	3.36 (2.48)	5.13 (2.81)	<0.0001
ES log (mean ± SD)	3.9 (4.2)	7.2 (6.8)	<0.0001
Peripheral arterial disease (*n*. %)	43/553 (7.8%)	44/228 (19%)	<0.0001
Renal failure (*n*. %)	13/553 (2.4%)	21/228 (9.2%)	0.0004
CPB time (minutes. Mean ± SD)	50 (15)	50 (15)	0.8031
Operation time (minutes. Mean ± SD)	153 (56)	150 (26)	0.8031
LVEF day after surgery %	53 (9)	47 (11)	<0.0001
CK-MB after 6 h	37 (20)	43 (38)	0.5946
CK-MB after 12 h	42 (32)	53 (70)	0.2007
CK-MB after 24 h	44 (35)	59 (100)	0.1307
Intraaortic balloon pump (*n*. %)	2/548 (0.4%)	2/227 (0.9%)	0.98331
Milrinone (*n*. %)	11/541 (2.0%)	9/222 (4.1%)	0.48196
TIA (*n*. %)	5/551 (0.9%)	0/227 (0%)	0.63631
Post-operative myocardial infarction (*n*. %)	6/551 (1.1%)	7/227 (3.1%)	0.27267
Post-operative stroke (*n*. %)	2/551 (0.4%)	5/227 (2.2%)	0.13234
Post-operative atrial fibrillation (*n*. %)	119/551 (22%)	58/227 (26%)	0.55622
Infection (*n*. %)	30/559 (5.4%)	39/228 (17%)	<0.0001
Infection source (*n*. %)			<0.0001
0	529/559 (95%)	189/228 (83%)	
BSI	6/559 (1.1%)	9/228 (3.9%)	
SSI sternal	9/559 (1.6%)	6/228 (2.6%)	
SSI leg	2/559 (0.4%)	1/228 (0.4%)	
Pneumonia	13/559 (2.3%)	23/228 (10%)	
Post-operative drainage (mL. mean ± SD)	552 (353)	530 (306)	0.9020
Red blood cells (mL. mean ± SD)	124 (271)	181 (326)	0.0363

LVEF—left ventricular ejection fraction, ES—EuroSCORE, ES log—logistic EuroSCORE, NYHA—New York Heart Association, TIA—transient ischemic attack, CPB—cardiopulmonary by-pass, CK-MB—creatine kinase-myocardial band, BSI—blood stream infection, SSI—surgical site infection.

## Data Availability

All data will be available for reasonable request.
